# Assessment of therapeutic efficacy and safety of artemether-lumefantrine (Coartem®) in the treatment of uncomplicated *Plasmodium falciparum* malaria patients in Bahir Dar district, Northwest Ethiopia: an observational cohort study

**DOI:** 10.1186/s12936-015-0744-x

**Published:** 2015-06-05

**Authors:** Yehenew A. Ebstie, Ahmed Zeynudin, Tefera Belachew, Zelalem Desalegn, Sultan Suleman

**Affiliations:** School of Medicine, Addis Ababa University, PO Box 9086, Addis Ababa, Ethiopia; School of Medical Laboratory and Pathology, College of Public Health Medical Sciences, Jimma University, Jimma, Ethiopia; Department of Population and Family Health, College of Public Health Medical Sciences, Jimma University, Jimma, Ethiopia; School of Pharmacy, College of Public Health Medical Sciences, Jimma University, Jimma, Ethiopia

**Keywords:** Artemether, Lumefantrine, Coartem®, Efficacy, Malaria, Parasitaemia, Haemoglobin

## Abstract

**Background:**

Malaria is a complex disease, which varies in its epidemiology and clinical manifestation. Although artemether-lumefantrine has been used as first-line drug for uncomplicated *Plasmodium falciparum* malaria in Bahir Dar district since 2004, its efficacy has not yet been assessed. The main objective of this study was to quantify the proportion of patients with uncomplicated falciparum malaria who were prescribed artemether-lumefantrine and who failed treatment after a 28-day follow-up.

**Methods:**

The research team attempted to conduct an observational cohort study on the assessment of therapeutic efficacy and safety of artemether-lumefantrine in falciparum malaria patients aged over five years in Bahir Dar district from March to July 2012.

**Results:**

Among 130 participants in the study, 60 % were males with 1:5 male to female ratio. The mean of asexual parasitaemia load was 8675 parasites/μL and 96.1 % participants were free from parasitaemia at day 3. At the end of the study, 98.5 % of participants showed adequate clinical and parasitological response of the drug. In the study, only 1.5 % of participants were shown late parasitological failure between seventh and 14th day follow-up and 1.3 % of participants were free from anaemia at the end of follow-up.

**Conclusion:**

According to the research findings, artemether-lumefantrine fulfilled the inclusion criteria of WHO as first-line drug and continues to be the drug of choice for the treatment of uncomplicated falciparum malaria. Outputs from this study should be supported through advanced molecular techniques and blood concentration and pharmaco-vigilance of the drug.

## Background

Malaria remains an important public health concern all over the world. According to a World Health Organization (WHO) report, the global incidence of the disease was 402 million in 107 malaria-endemic countries, and among those, 57 % of cases occurred in sub-Saharan Africa [1, 2] and 72 % of African cases were due to *Plasmodium falciparum* [3]. According to the Ethiopian Ministry of Health Report 2011, malaria caused 14 % outpatients and 9 % health facility admissions in 2009/2010 [4, 5].

Artemether-lumefantrine (Coartem®) (AL) is a widely accepted artemisinin-based combination therapy (ACT) due to the emergence of resistance to previously commonly used drugs, such as chloroquine and sulphadoxine-pyrimethamine [6–10]. After the implementation of AL as the drug of choice, a radical reduction in malaria cases occurred in most African countries: Rwanda (64 %), Zambia (29 %) and Ghana (13 %) between 2000 and 2007 [11, 12]. AL was introduced in Ethiopia as the drug of choice in 2004 and the disease morbidity and mortality rate decreased by 60 and 51 %, respectively, in 2005 and 2007 [13].

According to WHO criteria, approval of any anti-malarial drug as a first-line drug, it should have 90 % and above parasitological and clinical curative rates [13]. Many studies have indicated that AL is a drug which fulfills WHO requirements of a drug of choice for the treatment of uncomplicated malaria [14, 15]. However, studies conducted in Zanzibar, Thailand and Japan show treatment failure and the evolving of drug-resistant parasite gene *pfmdr1* 86 N for the drug [11, 16, 17]. Therefore, the purpose of this study was to determine the degree to which episodes of uncomplicated falciparum malaria, in patients age five years and above, had been treated with a recommended anti-malarial regimen in the area.

## Methods

### Study area and period

A 28-day observational cohort study was conducted on AL treatment effectiveness and safety, according to the 2009 WHO protocol [18], in Bahir Dar district, the capital of Amhara National Regional State, from March to July 2012. The city is located in northwest Ethiopia, 578 km from Addis Ababa. According to the Ethiopian Central Statistics Agency Report, total population of the city was 180,094, of which 87,089 were males [19]. In the area malaria is one of the ten top diseases and shows seasonal variation: high malaria cases are registered from September to December (end of summer) and from May to June (beginning of summer) every year [4].

### Recruiting of study participants

In the study 778 febrile cases screened for signs and symptoms of malaria by going house to house to recruit enough participants within a given time. Health extension workers and clinical nurses were mobilized to recruit febrile patients (patients with body temperature ≥37.5 °C), who were sent to the nearby health centre (Bahir Dar Han Health Center) with detailed information, including code number, sex, age, and date of recruitment. Patients were re-examined at the health centre for malaria symptoms, body temperature and weight. Screened individuals were sent to the laboratory department for blood film and haemoglobin determination. All laboratory procedures and tests were carried out by experienced laboratory technicians and blood collection was done aseptically. Patients who were *Plasmodium* positive and met the enrolment criteria, with full consent, were considered study participants.

### Treatment and follow-up

Six doses of AL (Coartem®, 20 mg artemether/120 mg lumefantrine) tablets were given for all study participants: who were positive for *P. falciparum* mono-infection; were five years or older and able to swallow the drug; who did not have severe acute malnutrition; had initial parasite densities between 1000 and 100,000 parasites/μl; had no other signs of severe and complicated falciparum malaria (i.e., impaired consciousness, respiratory distress, multiple convulsions, circulatory collapse, pulmonary oedema, abnormal bleeding, jaundice and haemoglobinurea); who took no anti-malarial drugs prior to the beginning of the study; and, had signed the consent form.

### Drug administration

Three days of drug were administered at the health centre based on age/body weight, under direct supervision of the research team, and participants were observed for at least 30 min for the presence of vomiting. Patients who vomited were retreated and re-observed for another 30 min, and if they were vomited again they were excluded and referred to the local hospital for further management.

### Appointing participants

Participants who finished their drugs properly were asked to come to the health centre on days 3, 7, 14, 21, and 28. When participants did not feel well, they were encouraged to comeback at anytime for advanced care. Participants who failed to come back to the health centre within the fixed schedules were visited by the research team on the same or the next day.

### Laboratory procedures

Two senior microscopists from Han Health Centre and one from Amhara Regional Laboratory personnel read the blood films and did haemoglobin determination. Capillary blood samples were collected aseptically and labelled anonymously (i.e., code number, days of follow-up and date). Thick and thin blood films were prepared at day 0, stained with freshly prepared 10 % Giemsa stain and examined for the loyalty of the inclusion criteria. Thick blood films were examined on days 2, 3, 7, 14, 21, 28, or any day patients returned spontaneously. Parasite count was done based on the number of asexual parasites observed against 200 leukocytes and multiplied by 40, to gain an approximate count per μl of blood. A slide was considered negative when no parasites were seen after examining 100 fields.

$$ \mathrm{Parasite}\ \mathrm{density}\ \left(\mathrm{per}\upmu \mathrm{l}\right) = \frac{\mathrm{Average}\kern0.5em \mathrm{number}\kern0.5em \mathrm{of}\kern0.5em \mathrm{parasites}\kern0.5em \mathrm{counted}\kern0.5em \times \kern0.5em \left(6000\hbox{-} 8000\right)\kern0.5em \mathrm{WBCs}}{\mathrm{Average}\kern0.5em \mathrm{Number}\kern0.5em \mathrm{of}\kern0.5em \mathrm{leukocytes}\kern0.5em \mathrm{counted}} $$

Haemoglobin value was measured based on packed cell volume method at days 0, 14 and 28 according to WHO 2009 protocol.

### Outcomes of the study

The effectiveness and safety of the drug was determined based on WHO protocol and classified as late parasitological failure, adequate response and therapeutically safe. Individuals were categorized as anaemic when their haemoglobin level was below 7 g/dl.

### Quality assurance

Three qualified microscopists (two from Han Health Center and one from Amhara Regional Laboratory department) read and counted parasite densities and any discordance on parasitic load calculation was done by averaging the two closest counts. In addition, all positives and 10 % randomly selected negative slides were cross-checked by the Regional Laboratory personnel.

### Data analysis

Data were reviewed and entered in to EPI Version 2002 statistical package and transformed and analysed by SPSS Version 16.0 package. Obtained data were evaluated by frequency, Chi-square (*χ*2) and logistic regression analyses, and a significance level of 5 % (α = 0.05) was established.

### Ethical approval

The research was ethically cleared by the Ethical Review Board of Jimma University and written informed consent from each participant or guardians of under-age children was obtained. All non-participant malaria patients were treated accordingly.

## Results

Between March and July 2012, 778 febrile cases, clinically suspected to be infected with malaria, were screened for eligibility. Of these 164 (21.1 %) had malaria-positive slides, 145 (18.6 %) and 11 (1.4 %) were positive *P. falciparum* and *Plasmodium vivax,* respectively, and the rest 0.9 % positive slides with mixed infections. Among 145 *P. falciparum* mono-infected patients, 10 could not be enrolled in the study due their ages and having a parasite load below 1000 parasites/μl of blood.

**Fig. 1:**
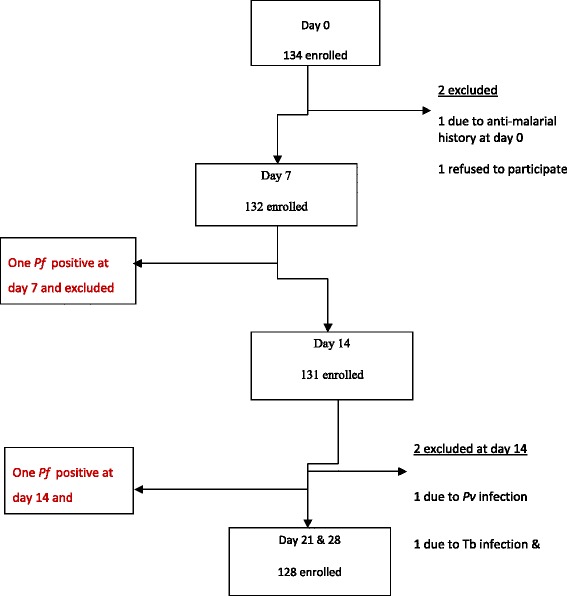
Details on study inclusion and follow up progress Bahir Dar district, March to July 2012. Among 778 malaria suspected and screened individuals, 134 *P.f.* mono-infected participants considered as eligible study subjects. Of them, 4 individuals excluded from the study and 130 study participants finished and drug efficacy and safety outcomes were drawn

Finally, 134 eligible patients enrolled in the study. As Fig. 1 shows, two were excluded (one took anti-malarial drug and another denied to participate). On day 7, 132 slides from patients were examined and one person was found to be *P. falciparum* positive and excluded as treatment failure and treated with quinine. On day 14, 131 slides were examined and three were found positive: one for *P. falciparum* and excluded as a treatment failure after treated with chloroquine, the second and third participants were positive for *P. vivax* and tuberculosis and excluded from the study. Slides from the remaining 128 patients were examined for Day 21 and 28.

**Fig. 2:**
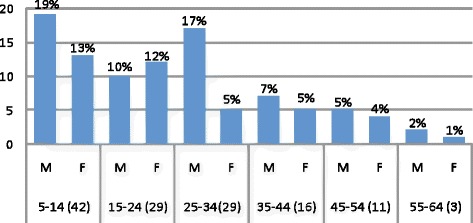
Demographic characters of study subjects within 28 days of follow-up, Bahir Dar district, March to July 2012. A total of 130 eligible participants, 78 (60 %) males with 1:5 male to female ratio. The majority of study subjects (32.33 %) were in age group five to 14 years and males were in the majority in the age groups

As Fig. 2 shows, majority (60 %) of participants were males and majority of participants (32.3 %) were in age group five to 14 years. The mean body weight and auxiliary temperature of participants at day 0 were 40.3 kg and 38.6 ± 0.18 °C, respectively.

Table 1 shows that, the mean of asexual parasitaemia among 130 participants at day 0 was 8675 parasites/μL and 85.9 and 96.1 % participants were free from parasite at days 2 and day 3, respectively. Interestingly, no gametocyte stages were detected at day 1 and only 3 % of participants were identified with a mean of 1800/μl gametocyte stages at day 3 and cleared totally at day 7. This study didn’t show any significant association between age differences and parasitic clearance (*P* = 0.877, 95 % CI) and only 1.5 % (2/130) of participants showed late parasitological failure in between the seventh and 14th day of follow-up and at the end, 98.5 % participants showed adequate clinical and parasitological response as Table 2 shows.

**Table 1:** Baseline characteristics of participants stratified by age and sex, Bahir Dar district, March to July 2012

Age group in yrs, (n)	Sex		Mean weight (kg)	Average body temp. (°C)	Mean Hgb (g/dl)	Geo. mean of parasitic load (/μl)
5–14 (42)	M	25	19.44	38.56	9.65	4959.15
	F	17	21.03	38.41	10.6	7313.63
15–24 (29)	M	14	44.65	38.67	7.4	6082.76
	F	15	36.72	38.41	10.71	4830.22
25–34 (29)	M	22	53.47	38.73	10.9	6983.98
	F	7	51.57	38.97	10.74	10,606.5
35–44 (16)	M	9	62.56	38.71	11.81	7502.47
	F	7	51.29	38.44	11.2	10,477.14
45–54 (11)	M	6	62.07	39.05	12.48	7420
	F	5	48.40	39.12	11.88	14,960
55–64 (3)	M	2	50.10	38.3	9.35	10,950.34
	F	1	47	38.4	13.4	12,017.07

The mean weight and auxiliary temperature among 130 participants at day 0 were 40.31 kg and 38.64 ± 0.18 °C, respectively. The average geometric mean of parasitic load and mean of haemoglobin value at day 0 were 8675.27 parasites/μL and 10.84 g/dl, respectively

**Table 2:** Efficacy outcome of AL stratified by age, Bahir Dar district, March to July 2012

Outcome	Age group					Total
	5–14 (42)	15–24 (29)	25–34 (29)	35–44 (16)	≥45 (14)	
	No. (%)	No. (%)	No. (%)	No. (%)	No. (%)	No. (%)
ETF	0	0	0	0	0	0
LCF	0	0	0	0	0	0
LPF	0	0	1 (0.8)	0	1 (0.8)	2 (1.56)
ACPR	42 (100)	29 (100)	28 (99.2)	16 (100)	13 (99.2)	128 (98.4)
Total analysis	42	29	29	16	14	130
Withdrew	2		0	2	0	5
Total	44	29	29	18	14	134

*ETF* Early treatment failure, *LCF* Late clinical failure, *LPF* Late parasitological failure, *ACPR* Adequate clinical and parasitological response, *With* withdrew, *No* Number, *%* percent

**Fig. 3:**
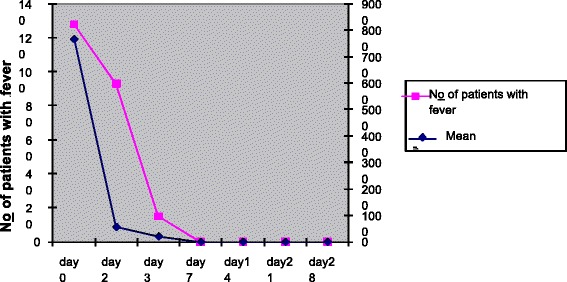
Summary of fever and parasitic load clearances within 28 days of follow-up, Bahir Dar district, March to July 2012. The study tried to correlate the overall febrile case reduction with the geometric mean of parasitic clearance at days 0 and 3. Fever clearance rate was insignificantly associated with parasitic clearance (P = 0.054, 95 % CI)

Some 98.46 % participants showed fever body temperature (>37 °C) at day 0 which lowered to 12.13 % at day 3, however, decrease in febrile cases was insignificantly associated with parasitic clearance (*P* = 0.054, 95 % C.I) as Fig. 3 shows.

At the end of the study, the mean haemoglobin level was increased from 10.82 g/dl to 11.21 g/dl as Table 3 shows and 1.3 % anaemic participants became non-anaemic (≥11 g/dl). Mean haemoglobin value was inversely correlated with decline in parasitic load (R = −0.430, 95 % CI) and the occurrence of anaemia was not associated with age differences [*X*^2^ tab (α = 0.05,df = 4) = 6.42].

**Table 3:** Summary of the average heamoglobin concentration (g/dl) stratified by age

Age group (years)						Total Hgb (g/dl)
Days	5–14 (42)	15–24 (29)	25–34 (29)	35–44 (16)	≥45 (14)	
	Hgb	Hgb	Hgb	Hgb	Hgb	
Day 0	10.11	11.08	10.17	12.52	10.24	10.82
Day 14	10.28	10.71	10.39	12.7	12.14	11.22
Day 28	10.63	10.71	10.96	11.74	12.02	11.21

Mean haemoglobin value was increased from 10.82 g/dl at day 0 to 11.21 g/dl at the end of the study

*Hgb* haemoglobin

Overall, only 32 % of study participants showed common and typical types of adverse effects and the most frequently reported adverse events were weakness/fatigue and headache (15.9 %), mouth rash (14.7 %), cough (11.5 %) and joint pain (2.6 %) and loss of appetite (1.3 %) were the least frequent adverse effects (Table 4). However, the presence of any side effects was statistically insignificant with age and sex (*P* > 0.15, 95 % CI).

**Table 4:** Percent distribution of adverse effects of AL among participants in each age group and sex, Bahir Dar district, March to July 2012

Adverse effects	Sex	Age group in years (%)					Total (130)
		5–14 (42)	15–24 (29)	25–34 (29)	35–44 (16)	45+ (14)	
Weakness	M	24	10	16	6	0	15.9
	F	8	14	4	4	0	
Headache	M	24	16	20	10	0	15.9
	F	12	9	0	0	0	
Mouth rash	M	10.4	8	15	17.4	0	14.7
	F	13.0	21.7	0	4.4	0	
Cough	M	7.2	12.7	16. 7	0	0	11.5
	F	13.1	16.7	0	0	0	
Abdominal pain	M	21.4	21.4	21.4	0	7.14	8.9
	F	0	21.4	7.1	0	0	
Sore throat	M	11.3	7.1	0	5.2	0	8.9
	F	21.4	15.7	0	7.1	0	
Tongue Inflammation	M	6.1	9.2	0	9.1	0	7.01
	F	7.3	16.4	0	0	0	
Joint pain	M	9.8	0	0	10	0	2.6
	F	0	6	0	0	0	
Loss of appetite	M	0	0	0	8	0	1.3
	F	0	0	0	0	0	

All adverse effects seen in the study were registered under FDA as the usual type side effects. In the study the most frequents were weakness and headache followed by mouth rash. Whereas, loss of appetite and joint pains were the least among others

## Discussion

According to WHO recommendation, selection of an anti-malarial drug as a drug of choice, it should have 90 % and above parasitological and clinical cure rates [13]. Since adoption of AL as the drug of choice, many studies have been conducted and its high parasitological and clinical curatives capacity fulfills WHO selection criteria. According to this study, total efficacy result of the drug was 98.3 % which is in line with WHO recommendation and researches conducted in Ethiopia, Thailand and elsewhere [11, 14, 15, 20].

AL is a drug with a six- to eight-fold ability to decrease asexual and gametocyte stages than older anti-malarial drugs [21, 22]. Parasitaemia was reduced by 60 and 96.1 % at days 2 and 3, respectively, and totally cleared at day 7 of follow-up. Research conducted in Ethiopia, Mali, Zambia, Thailand and Uganda has shown the maximum parasitaemia clearance time was at the seventh day of study period [11, 15, 20, 23, 24].

The observed adverse effects of AL in the study were similar between adults and children and registered under the food and drug administration authority (FDA). The most common adverse events were cough, upper respiratory tract inflammation and abdominal pain, which were comparable to findings conducted in Ethiopia, Uganda and Mali [15, 20, 23].

At the end of the study, anaemic cases decreased by 1.3 % as similar studies reported in Angola and Mali [23, 25]. A total mean value of haemoglobin was insignificantly associated with decline of parasitic load (*P* > 0.05, 95 % CI), which might be due to the high prevalence of other intestinal parasitosis in the area; it is known that anaemia is the most prevalent, with hookworm and other intestinal parasite-infected areas [26, 27].

## Conclusion

This finding revealed that AL is safe and effective and thus continues to be the drug of choice for the treatment of uncomplicated falciparum malaria as first-line drug. No severe adverse effects were recorded other than FDA registered. The six-dose regimen of AL is safe and well tolerated drug for the treatment of acute falciparum malaria in the area.

All stakeholders in malaria treatment and management should have determination on promoting rational use of medication at every step to sustain the efficacy and safety of the drug. Post-marketing drug surveillance of AL (Coartem®) should be done for monitoring and evaluation of the quality, safety and efficacy of the drug. Further PCR-based effectiveness studies with pharmaco-vigilance of the drug should be undertaken, to prevent decreases in drug adherence which can lead to the development of drug resistance.

## Abbreviations

ACPR: Adequate clinical and parasitological response
ACT: Artemisinin-based combination therapy
AL: Artemether-lumefantrine
ANRS: Amhara national regional state
ETF: Early treatment failure
FDA: Food and drug administration
LCF: Late clinical failure
LPF: Late parasitological failure
WHO: World Health Organization
With: Withdrew
No: Number
